# Supplementary figures and images for: Fossil Sirenia from the Pleistocene of Qatar: new questions about the antiquity of sea cows in the Gulf Region
Source: PeerJ. 2022 Oct 18;10:e14075. doi: 10.7717/peerj.14075 (PMC9586076; doi:10.7717/peerj.14075)

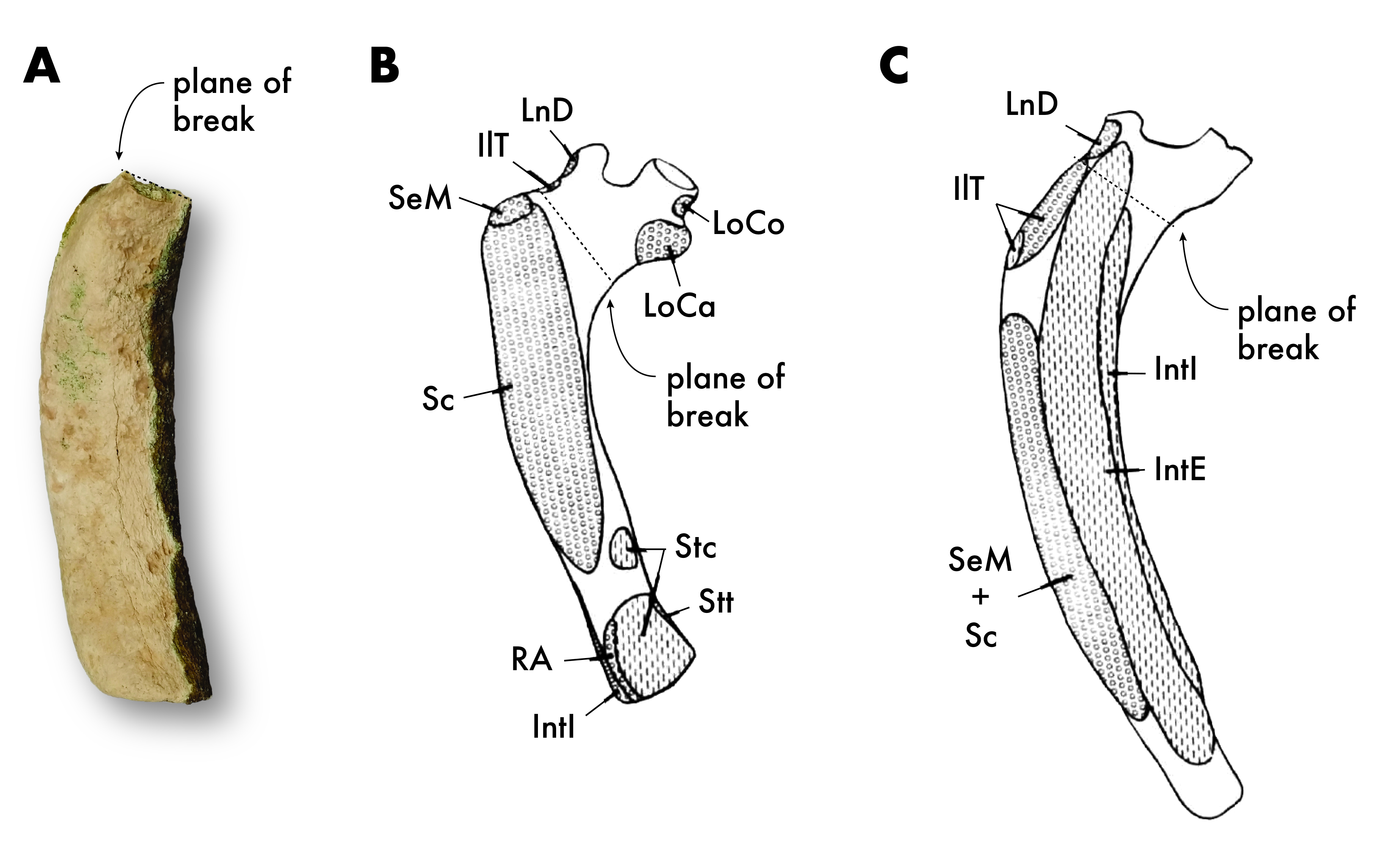

Supplement: Supplemental Information 1 — QM.2021.0504, Dugongidae right first (?) rib (A), compared with the osteology and myology of the first (B) and second (C) ribs in Dugong dugon, reflected from (Domning, 1977: Figs. 41 and 42). Abbreviations: IntE and IntI, mm. intercostalis externi and interni; IlT, m. iliocostalis thoracis; LoCa, m. longus capitis; LoCo, m. longissimus colli; LnD, m. longissimus dorsi; RA, rectus abdominus Sc, m. scalenus; Se, m. serratus; Stt, m. sternohyoideus; StC; m. sternocostalis. [file peerj-10-14075-s001.jpg]
