# Supplementary material for: Fossil Sirenia from the Pleistocene of Qatar: new questions about the antiquity of sea cows in the Gulf Region
Source: PeerJ. 2022 Oct 18;10:e14075. doi: 10.7717/peerj.14075 (PMC9586076; doi:10.7717/peerj.14075)
Supplement: Supplemental Information 2 — Includes description of reports in the gray literature, references, and correspondence regarding collecting authorization. [file peerj-10-14075-s002.docx]

**Supplemental Information**

**Fossil Dugongid from the Pleistocene of northern Qatar: new questions about the antiquity of sea cows in the Gulf Region**

Nicholas D. Pyenson^1,2^, Mehsin Al Ansi^3^, Clare M. Fieseler^1^, Khalid Hassan Al Jaber^4^, Katherine D. Klim^1^, Jacques LeBlanc^5^, Ahmad Mujthaba Dheen Mohamed^3^, Ismail Al Shaikh^6^, Christopher D. Marshall^7,8^

^1^Department of Paleobiology, National Museum of Natural History, Smithsonian Institution, Washington, DC 20059, U.S.A.

^21^Department of Paleontology and Geology, Burke Museum of Natural History and Culture, Seattle, WA, 98105, U.S.A.

^3^Department of Biological and Environmental Sciences, Qatar University, Doha, State of Qatar

^4^National Museum of Qatar, Doha, State of Qatar

^5^Consulting Geologist, Calgary, Alberta, Canada

^6^ExxonMobil Research Qatar, Qatar Science and Technology Park - Tech 2, Doha, State of Qatar

^7^Department of Marine Biology, Texas A&M University Galveston Campus, 200 Seawolf Parkway, Galveston, TX 77554, USA

^8^Department of Ecology and Conservation Biology, 2258 TAMU, Texas A&M University, College Station, TX 77843, USA

Corresponding Author:

Nicholas D. Pyenson^1^

Smithsonian Institution, MRC 121, Natural History Building, West Loading Dock, 10th Street Northwest & Constitution Ave. NW, Washington, DC 20560 USA

Email address: [pyensonn@si.edu](mailto:pyensonn@si.edu)

**Supplemental Dataset**

We provide a supplemental dataset of first and second rib measurements from fossil and extant sirenians, including abbreviations. See main text for museum collection abbreviations. All measurements in mm.

**Other fossil Dugongidae from Qatar**

LeBlanc (2009, 2015, 2017) provided the first report of fossil sirenian material from Qatar, including the description of fossil Dugongidae from the Early Miocene age Dam Formation of southwestern Qatar, and fossil sirenians from the Eocene age Dammam Formation of western Qatar. This material mostly consists of postcranial elements from occasionally associated individual skeletons with complete (or partially complete) individual elements, such as ribs and, less commonly, vertebral elements.


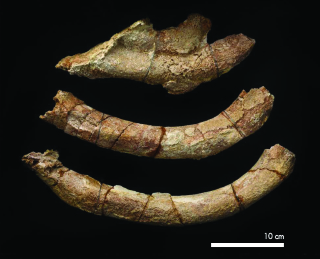


Some of this material, including Dam Formation dugongid ribs bones (QNM.2011.660.2ABC, modified from Kaskes and Baastians, unpublished data, above), is on display in the galleries of the National Museum of Qatar. QNM.2011.660.2ABC likely represents three anterior ribs from an Early Miocene dugongid (gen. indet.), with similarities to the Pleistocene age rib fragment QM.2021.0504, as mentioned in the main text. The full list of specimens from the Dam and Dammam formations in the collections of the National Museum of Qatar in Doha includes: QNM.2011.660.2ABC, QNM.P.2017.2005, QNM.P.2017.2006, QNM.P.2017.2007, QNM.P.2017.2008, QNM.P.2017.2009, ARC.2008.14.387, ARC.2008.14.388, and ARC.2008.14.389, QM.2021.0504.

**Gray Literature References**

LeBlanc J. 2009. A Fossil Hunting Guide to the Miocene of Qatar, Middle East: A Geological & Macro-Paleontological Investigation of the Dam Formation. 192 pages. DOI: 10.13140/RG.2.1.1228.6567 (self-published online)

LeBlanc J. 2015. A historical account of the stratigraphy of Qatar, Middle East (1816 to 2015). 1220 pages. DOI: 10.13140/RG.2.1.5165.6725 (self-published online)

LeBlanc J. 2021. Stratigraphic Lexicon: A revised guide to the Cenozoic Surface Formations of Qatar, Middle East (excluding the islands). *Biosis: Biological Systems* 2(4), 361–407.

Kaskes P, Bastiaans D. 2017. Exhibiting the Geological History of Qatar: Fieldwork and Collection Report for Gallery 1 of the National Museum of Qatar. Unpublished report, version 3. Naturalis Biodiversity Center, Leiden, Netherlands. 150 pp.
